# Supplementary material for: Cooling phase organic fertilizer applied in saline soil increase the content of soil macro-aggregates and aggregate-associated carbon
Source: PLoS One. 2025 Sep 17;20(9):e0330756. doi: 10.1371/journal.pone.0330756 (PMC12443260; doi:10.1371/journal.pone.0330756)
Supplement: Attachment — Submitted filename: Response to Reviewers.docx [file pone.0330756.s003.docx]

**Response to Reviewers**

**Manuscript Title: Cooling Phase Organic Fertilizer Applied in Saline Soil Increase the Content of Soil Macro-aggregates and Aggregate-associated Carbon**

**Manuscript number: PONE-D-24-51544**

We would like to sincerely thank the editor and all reviewers for their careful and thorough review of our manuscript and for providing valuable comments and suggestions, which have helped us to improve the quality of our manuscript. We have made a point - by - point response to the reviewers' comments below.

Reviewer 1's Comments and Responses

**Reviewer's comment 1:** Abstract, please add more information about methods, then, please add some key data for some contents of results, for example: Compared with CK after 180 days, the four fertilization treatments significantly increased the contribution rate of organic carbon to each soil aggregate, among which the contribution rate of organic carbon to macro-aggregate was significantly lower in mature compost treatment, and the contribution rate of organic carbon to micro aggregates was relatively higher.
Response: Thank you for pointing this out. We agree with this comment. Therefore, we make the following changes [ We have re-summarized and rewritten the abstract ]

**Reviewer's comment 2:** Introduction, please give more contents about SOC in soil in the Yellow River basin, meanwhile, please add the condition about organic fertilizer application in local area.

Response: Thank you for pointing this out. We agree with this comment. Therefore, we make the following changes [ We have added relevant content in lines 57 and 74 ]

**Reviewer's comment 3:** Test Material, please give the soil classification name by FAO or others.

Response: Thank you for pointing this out. We agree with this comment. Therefore, we make the following changes [ We have added relevant content in line 101 ]

**Reviewer's comment 4:** Results and Analysis, there were seven tables, however, none figure, so, please change some tables to figures. Otherwise, table 2 was simple, please delete it and use words in MS.

Response: Thank you for pointing this out. We agree with this comment. Therefore, we make the following changes [ We modified Table 5 and Table 6 to Figure 2 and Figure 3 respectively. Table 2 has been expressed in text form ]

**Reviewer's comment 5:** Discussion, according to the results of this study, how should organic fertilizer be applied to promote soil fertility improvement? Please further study or provide suggestions.

Response: Thank you for pointing this out. We agree with this comment. Therefore, we make the following changes [ We have added potential future research directions to the discussion (Lines 324, 357) and summarized a set of organic fertilizer application methods that can improve soil fertility in the conclusion ]

**Reviewer's comment 6:** Conclusion, please shorten.

Response: Thank you for pointing this out. We agree with this comment. Therefore, we make the following changes [ We revised and streamlined the conclusion ]

Reviewer 2's Comments and Responses

**Reviewer's comment 1:** The abstract contains multiple grammatical errors and improper use of tenses, making it difficult to read. A thorough English language polishing is strongly recommended.

Response: Thank you for pointing this out. We agree with this comment. Therefore, we make the following changes [ We rewrote the abstract and carefully checked the grammar and tense issues ]

**Reviewer's comment 2:** The title focuses on "cooling phase organic fertilizer," but the introduction spends a lot of space discussing general soil aggregates and organic carbon without emphasizing why the "cooling phase" is special or worth studying.

Response: Thank you for pointing this out. We agree with this comment. Therefore, we make the following changes [ We have revised the introduction, which presents the physicochemical and microbial characteristics of organic fertilizers during the cooling period and thus reflects the research value of organic fertilizers in this period. Lines 50-70 ]

**Reviewer's comment 3:** Sentences comparing treatments are often confusing. Changes over "0-180 days" and "180-540 days" are mentioned, but without a clear, structured presentation.
Response: Thank you for pointing this out. We offer the following explanations [ The 10-180d data refers to the increment of Bulk Density from the 10th day to the 180th day. The 180-540d data refers to the increment of Bulk Density from the 180th day to the 540th day ]

**Reviewer's comment 4:** The discussion mainly describes the observed changes but lacks deeper explanations of underlying mechanisms.
Response: Thank you for pointing this out. We agree with this comment. Therefore, we make the following changes [ We resummarized and rewrote the discussion, adding some more in-depth explanations of the underlying mechanisms ]

**Reviewer's comment 5:** The conclusion currently serves as a summary rather than a synthesis. Please deepen it to highlight the broader significance and potential implications.

Response: Thank you for pointing this out. We agree with this comment. Therefore, we make the following changes [ We re-summarized and refined the conclusion and proposed a set of fertilization strategies ]
